# Evolving Concepts of Dyslexia and Their Implications for Research and Remediation

**DOI:** 10.3389/fpsyg.2019.02873

**Published:** 2019-12-18

**Authors:** Athanassios Protopapas

**Affiliations:** Department of Special Needs Education, University of Oslo, Oslo, Norway

**Keywords:** literacy, reading difficulties, reading diagnosis, reading failure, dyslexia

## Abstract

Aspects of dyslexia definitions are framed as a contrast between the past and the future, focusing on implications for research and remedial education, highlighting assumptions that bias or limit research or clinical practice. A crucial development is evident in understanding dyslexia, moving from its conceptualization as a discrete identifiable condition toward the realization of continuity with the general population with no clear boundaries and no qualitative differences. This conceptual evolution amounts to a transition from considering dyslexia to be some entity that causes poor reading toward considering the term dyslexia to simply label poor reading performance. This renders obsolete any searches for abnormalities and directs efforts toward understanding reading skill as a multifaceted domain following a complex multifactorial developmental course.

## Introduction

In this paper, I discuss aspects of definitions of dyslexia framed as a contrast between the recent past – a few decades – and the near future – the next decade or so. I only consider what I judge to be “primary” definitions, aimed at providing a conceptual understanding, rather than diagnostic criteria or guidelines aimed at practical clinical application (e.g., DSM-V: American Psychiatric Association [Apa], 2013 and ICD-11: World Health Organization, 2018).^1^ Such clinical guidelines are secondary to conceptual definitions in the sense that they can only express attempts to operationalize concepts as understood by a community of researchers and/or practitioners. I then focus on implications, because that is where it matters most, and also because definitions reflect assumptions that may bias or limit our research or clinical practice without our noticing. One valid approach to this topic might be to discuss specific definitions at some length, pointing out advantages and disadvantages, what they imply and how they can be applied. Instead of that, I take a more general look, abstracting away from specific definitions, and focusing on common themes that distinguish the past from the future.

First of all, why define dyslexia? What is the point of even having definitions? One reason might be to express our understanding. That is, once we know exactly what something is and is not, we can construct a definition that distils our understanding into a clear, concise statement of who belongs to the category and who does not. Applying such a definition would be straightforward and would indicate unambiguously whether an individual falls under the domain of the definition or not. An alternative reason might be seen in very different situations, when it is not clear at all how to distinguish members from non-members; when, despite our efforts, the situation remains complex, with obvious disadvantages to any proposals, and an impasse is reached. Then, we may try to impose a definition, in essence to command a somewhat arbitrary set of choices, just to be able to move forward. We would not normally do that, instead of trying harder to understand, but there are situations when practical needs of classification prevail, as in dyslexia: we need to be able to decide whether we should treat any particular individual child one way or another.

In the case of dyslexia, this issue has been extensively discussed. In a recent book that has attracted much attention, Elliott and Grigorenko (2014) listed a number of different “understandings of who may be considered to have dyslexia” (p. 39). For example, the term may refer simply to anyone who struggles with accurate single-word decoding; or to those with a more pervasive condition marked by various comorbid features and symptoms; or to those with a significant discrepancy between decoding and another measure such as IQ or listening comprehension; or to those with a certain cognitive profile associated with their reading difficulty; and there are several more possibilities. In other words, there is a very wide range of different uses of the term dyslexia, and associated definitions. So, as far as understanding goes, it seems fair to conclude, as Elliot and Grigorenko did, that although “it is incontrovertible that there is a significant number of individuals who struggle to learn to read, … achieving a clear, scientific, and consensual understanding of [the term dyslexia] has proven elusive” (p. 38). In other words, there is no clear understanding. So, our definitions of dyslexia are not meant to express understanding but, rather, to serve pressing practical needs.

What are these practical needs that require a definition of dyslexia? For research, we need to know how to form our research groups. That is, whom to study, in order to understand dyslexia, and whom to exclude. A definition also dictates what we should measure to document the group inclusion and to examine the features of the classification. Similarly, in education, we need to know which children are selected for remedial services, a decision of the utmost importance given that poor literacy is associated with poor academic, social, behavioral, emotional, professional, financial, and health outcomes (e.g., Goldston et al., 2007; Gross et al., 2009; Undheim et al., 2011; McLaughlin et al., 2014). The definition also directs our assessment and educational programs, by highlighting what needs to be assessed to justify the selection, to document the relevant educational needs, and also to guide the setting of specific objectives to be achieved by remedial education.

## Elements of Dyslexia Definitions

Table 1 lists a few selected definitions proposed over the past several decades up to the present time. Before considering the differences between past and future, let us begin with a common element weaving through our understanding of dyslexia across time, taking various specific forms in different approaches, but expressing an underlying unifying theme. This is the element of *unexpectedness* of the reading difficulty. That is, dyslexia is seen as a difficulty or inability to learn to read in a situation where we would have expected success. This is an important element because it reflects the notion that, in contrast to oral language, reading doesn’t just come naturally and universally (cf., Liberman, 1995). The idea is that there is some set of conditions under which we would expect children to learn to read, and for some children that does not happen.

**TABLE 1:** Selected definitions of dyslexia.

**Year**	**Definition**	**Organization**	**Source**
1968	[Dyslexia is] a disorder manifested by difficulty in learning to read despite conventional instruction, adequate intelligence and sociocultural opportunity. It is dependent upon fundamental cognitive disabilities which are frequently of constitutional origin	World Federation of Neurology	Critchley (1970), as cited in Snowling (2000, p. 15)
1994	Dyslexia is one of several distinct learning disabilities. It is a specific language-based disorder of constitutional origin characterized by difficulties in single word decoding, usually reflecting insufficient phonological processing abilities. These difficulties in single-word decoding are often unexpected in relation to age or other cognitive abilities; they are not the result of generalized developmental disability or sensory impairment. Dyslexia is manifested by a variable difficulty with different forms of language, including, in addition to a problem with reading, a conspicuous problem with acquiring proficiency in writing and spelling	Orton Dyslexia Society (now called the International Dyslexia Association)	Snowling (2000, pp. 24–25)
2002	Dyslexia is a specific learning disability that is neurobiological in origin. It is characterized by difficulties with accurate and/or fluent word recognition and by poor spelling and decoding abilities. These difficulties typically result from a deficit in the phonological component of language that is often unexpected in relation to other cognitive abilities and the provision of effective classroom instruction. Secondary consequences may include problems in reading comprehension and reduced reading experience that can impede growth of vocabulary and background knowledge	International Dyslexia Association	https://dyslexiaida.org/definition-of-dyslexia/ (downloaded March 2018)
2009	Dyslexia is a learning difficulty that primarily affects the skills involved in accurate and fluent word reading and spelling	British Dyslexia Association	https://www.bdadyslexia.org.uk/news/definition-of-dyslexia (downloaded December 2019)
	Characteristic features of dyslexia are difficulties in phonological awareness, verbal memory and verbal processing speed		
	Dyslexia occurs across the range of intellectual abilities		
	It is best thought of as a continuum, not a distinct category, and there are no clear cut-off points		
	Co-occurring difficulties may be seen in aspects of language, motor co-ordination, mental calculation, concentration and personal organization, but these are not, by themselves, markers of dyslexia		
	A good indication of the severity and persistence of dyslexic difficulties can be gained by examining how the individual responds or has responded to well founded intervention		
2017	[Dyslexia is] a persistent and unexpected difficulty in developing age- and experience-appropriate word reading skills	N/A	Parrila and Protopapas (2017)

Stated this way, the notion of unexpectedness is very vague. What is the basis for the expectations? This is a domain in which we see some evolution in our thinking, both in the kinds of conditions we consider conducive to reading, or necessary for reading, as well as in how the conditions are operationalized, sometimes forming exclusion criteria in a definition.

The first element contributing to expectation is age and experience. Clearly, a 4-year-old cannot have dyslexia, by definition. This is because at this age most children are not yet mature enough in their metalinguistic skills to be able to acquire reading. So, being unable to read at 4 years of age is normal, and expected. We also will not think that a child who cannot read has dyslexia if the child has not been sufficiently exposed to print or has not practiced reading. This is because reading skill does not develop spontaneously but requires substantial experience with print. So, a child who has not practiced reading is not expected to be able to read.

A second element concerns limitations arising from sensory perception. This expresses the intuitive notion that a child who cannot see well is not expected be able to read, because seeing the letters is obviously necessary for reading them. Similarly, though indirectly, a child who cannot hear well is not expected to have formed adequate representations of the speech sounds, that is, the phonemes, to which the letters map. Therefore, a hearing-impaired child is not expected to be able to learn to read well, at least not without extraordinary effort.

A third element concerns educational opportunity. This is not as straightforward to apply, but it is also based on the concept that reading does not develop spontaneously. So, we do not expect children to read unless they have been taught to read. This may mean that the child must at least attend school regularly, or have alternative instructional interactions to fulfill this role. In other approaches this element involves not only the existence of teaching but also the teaching method applied. Under this view, if children are not taught properly then we do not expect them to learn to read. So, a child who fails to make progress with one teaching approach, but then learns to read successfully using a different teaching approach, was not reading disabled in the first instance but, rather, “teaching-disabled” (Tunmer and Greaney, 2010).

Finally, general cognitive ability is an element commonly seen as related to the expectation of reading competence. This has taken various specific forms, some of which have led to great controversy. In the past, it was thought that at least average cognitive ability is necessary for learning to read, so we should not expect a developmentally disabled child to be able to read. This idea has not survived, because it has been shown that children with general intellectual disabilities, such as Down’s syndrome, can learn to read (Snowling et al., 2008; see also Næss, 2016), even though their understanding of what they read can only be commensurate with their general intellectual functioning. Nevertheless, some element of general cognitive ability is still included, in some form or other, in most approaches to dyslexia, even though the original notion of unexpectedness can no longer be sustained, perhaps for more practical reasons. For example, in education, cognitive ability can help distinguish children with dyslexia from children who cannot read successfully in the sense of not understanding what they read, which is a very different sort of reading failure (Catts et al., 2006; Nation and Angell, 2006). That is, the notion of dyslexia concerns the “mechanics” of reading, and not the comprehension of the written text, which is largely independent from word-level skills past the beginner stage (Bishop and Snowling, 2004; Nation, 2005). In research, an element of cognitive ability is included to ensure that participants in studies can respond similarly to the requirements of the tasks.

Beyond the common elements, which are seen in one form or another in most definitions, there are various additional components that are found in some approaches, but not all. For example, a 1994 definition from the Orton Dyslexia Society (today known as International Dyslexia Association) also included these terms: distinct, specific, constitutional, language-based, insufficient phonological processing, affecting single-word decoding, and difficulty with different forms of language, writing and spelling (Snowling, 2000, pp. 24–25; see Table 1). This approach was typical for its period. We can see that very different kinds of components were involved, ranging from assumptions about the nature of dyslexia to correlates and domains of symptoms. These are the kinds of things that have evolved over the decades. So, now we can turn to the discussion of such elements in a contrast between the past and the future.

## Progress in Understanding Dyslexia

As I mentioned earlier, we will not consider specific entire definitions. Rather, I will discuss elements found in definitions, as they express our attempts to understand what dyslexia is and to delineate what dyslexia is not. Let me start with the easier ones, in the sense that they seem to be mostly resolved already, having made much progress from the recent past, so that the future is likely going to be like the present in regard to them.

As already noted, the level of cognitive ability has played an important role in the concept of dyslexia. For a long time, and to some extent still seen in some places, an IQ discrepancy criterion has been applied, so that a child cannot be called dyslexic unless there is a substantial difference between general cognitive ability and reading skill. This was based on an expectation that intelligence determines word reading ability, which it does not; and on the assumption that intelligence makes a difference in how word-level reading difficulties should be remediated, which it does not (Elliott and Grigorenko, 2014). So the present approach, to a large extent, and the future approach, is that the concept of dyslexia is not defined against some IQ reference, and instead it is applied across ability ranges (Vellutino et al., 2004).

Another feature of the past is the list of various types of symptoms supposedly associated with dyslexia. This includes assortments of observations such as clumsiness, poor balance or poor sense of rhythm, left-handedness, ability to distinguish right from left, and so on. It also includes difficulties with other kinds of skills not directly related to reading, such as arithmetic, language, attention, visual and auditory perception, and others. For some of these there is evidence that they are found more frequently in poor readers than in typical readers; for others there is little empirical support (Ramus and Ahissar, 2012; Protopapas, 2014). But in any case these symptom lists cannot be part of the concept of dyslexia and have been, or are being, abandoned. The only skills that are relevant to the concept – and therefore the definition – of dyslexia are reading skills, at the word level (de Jong and van Bergen, 2017; although an argument can be made for a confirmatory role of testing specific well-attested reading-related skills; Pennington et al., 2012).

Speaking of reading skills, many definitions refer specifically to “decoding” skills, which concern processing of single words, and can be said to be sufficient when single words are pronounced correctly. This has proven to be insufficient in that word-level skills are not exhausted with accurate singe-word decoding, but also encompass reading fluency, which refers to the speed, or efficiency, of processing (Fuchs et al., 2001; Wolf and Katzir-Cohen, 2001) and, crucially, involves word sequences rather than isolated words (Protopapas et al., 2018; Altani et al., 2019). We must retain a distinction from comprehension, because comprehension concerns an altogether different set of skills and associated difficulties (Catts et al., 2006; Nation and Angell, 2006; Oakhill et al., 2014). But focusing on decoding alone is not the right point at which to draw the line, because it leaves out the efficient processing of word sequences, which precedes and facilitates comprehension. Rather, the line is to be drawn between reading comprehension, on the one hand, and both decoding of individual words and fluency in reading word sequences, on the other.

Turning to more abstract notions, a crucial development is evident in the concept of dyslexia as a discrete identifiable condition, most evident in educational and clinical settings (Elliott and Gibbs, 2008), moving toward the realization that there is no discrete condition but, rather, continuity with the rest of the population, with no clear boundaries and no qualitative differences. This is not a novel idea. The question of whether dyslexia makes up some special population, outside of the general distribution of reading skill, has been investigated for some time now. Over the past decades, it seems increasingly accepted that there is no distinct group and that dyslexia concerns the low end of the distribution of reading skill (Ahmed et al., 2012; Snowling, 2013; see Protopapas and Parrila, 2018, for more references).

Closely related to the notion of a discrete condition is the conceptualization of causal factors. The recent past, and indeed the present to a large extent, is dominated by views of dyslexia as caused by some specific factor. This makes sense if dyslexia is a specific condition. But once the notion of a qualitative distinction is discredited, the single-cause approach loses much of its appeal. And the same holds for theoretical attempts building on two – or even three – specific factors. Instead, recent evidence from genetic and behavioral analyses, and a reconceptualization of developmental courses, suggest that the roots of dyslexia are traced to many interacting factors at different levels of description, and that different routes can lead to similar outcomes, so that even a common difficulty is not necessarily attributable to a common history (Pennington, 2006; Snowling, 2011; van Bergen et al., 2014; see Parrila and Protopapas, 2017, for discussion).

In this context, it is instructive to consider another common element in definitions, namely the “constitutional” (or “neurobiological”) origin. This reflects the belief that one is born dyslexic, often taken to imply that one’s genes alone determine whether one will learn to read easily or not. If that were true, then pairs of identical twins, who have exactly the same genes, would always either both have dyslexia or both not have trouble learning to read. This is in fact not the case; more recent estimates of the concordance of monozygotic twin pairs in dyslexia seem to hover around 70%, as do estimates of heritability for reading skill measures (Grigorenko, 2004; Scerri and Schulte-Körne, 2010; Christopher et al., 2013; Bishop, 2015). This means that there is indeed a strong genetic component in propensity to acquire reading skills and in family risk for reading difficulties (Swagerman et al., 2017), at least within the fairly homogeneous environments in which such studies are typically conducted. But this should not be interpreted as implying that one is doomed by their genes. That is because, first, estimates of heritability in less homogeneously supportive environments are lower (Bishop, 2015). And second, because it is increasingly understood that the final behavioral outcomes, as noted above, depend on a multitude of interacting factors, only some of which concern genetics (Mitchell, 2018). Moreover, the way genetic factors are ultimately expressed involves environmental – hence pliable – circumstances, not limited to instruction but also potentially involving motivation, expectations, language, the creation of opportunities, the availability of resources, and more (van Bergen et al., 2014). In short, our understanding of what heritability means and how it should be interpreted is under major revision.

A related point is the characterization of dyslexia as a neurodevelopmental disorder, which is still seen often today (indeed in both main diagnostic manuals, DSM-V and ICD-11). Although it seems that some use this term to simply mean that many children have difficulties in learning to read because of how their brain is set up (rather than because of poor teaching, or poor character, for example), which is likely true, this is not an appropriate use of the term. According to the dictionaries, “disorder” in this sense is a medical term which means “a physical or mental condition that is not normal or healthy,”^2^ “a derangement or abnormality of function; a morbid physical or mental state,”^3^ “an illness that disrupts normal physical or mental functions,”^4^ “a disturbance of physical or mental health or functions.”^5^ In other words, it means “disease.” More specifically the term “neurodevelopmental disorder” is specifically associated with disrupted and impaired brain development (Bishop and Rutter, 2008; Thapar and Rutter, 2015), that is, a brain fault. This is unfortunate and inappropriate, turning a social and educational issue into a medical one. It is a view that will not prevail, in my opinion, because despite many efforts there are no indications of abnormality at any level of description, either genetic or neurological or cognitive or linguistic. There is much evidence confirming the existence of *differences between groups* of people with and without reading problems across some of these domains, but this kind of evidence is nowhere near establishing neurodevelopmental failure (see Protopapas and Parrila, 2018, for extensive discussion). Unfortunately, this issue is muddled by some researchers, who inappropriately use terms such as “neurological disorder” based on average differences between groups with and without dyslexia, with no evidence of disturbance or malfunction of the nervous system.

Importantly, documentation of group differences does not amount to evidence for abnormality. Consider a hypothetical comparison between top world-class violinists or gymnasts to “typical individuals”: it should be clear that some differences must exist in the structure and function of their central nervous systems, compared to the non-athletic or non-musical group, because it is only such neural differences that can account for the observed differences in athletic or musical performance. If the differences cannot be fully documented it is because our methods are still inadequate. So, in this sense it is trivial to document group differences in nervous system structure and function when differences in performance have been observed. This would not lead us to characterize the individuals with deviations from average performance as neurologically disordered. Likewise, group deviations – even extreme ones – from the average reading performance should hardly be characterized as “neurological disorders” but, rather, as expected occurrences in the context of individual variability (Protopapas and Parrila, 2018).

In other words, children with dyslexia are just a part of the general distribution, normal in all respects (except in response to literacy instruction), and their brains are within normal variability. The only domain in which a clear discrepancy is found is learning to read. However, reading is a cultural invention and a recent social development, which has led to educational systems and to the pressure for universal literacy. Of course these are all very positive developments, but this has nothing to do with the characterization of poor response to the cognitive demands of learning to read. Consider this analogy: If an otherwise perfectly normal child cannot learn to sing, or to compete in sports, despite substantial practice and efforts from teachers and coaches, would we consider that to be a neurodevelopmental disorder? I think not, and I think that this is where our understanding of dyslexia will take us – and should take us – in the near future. Of course the critical difference is that being clumsy or tone deaf has few, if any, consequences for life success, whereas poor reading is severely handicapping in the modern literate society, as noted above. Thus it is of crucial importance for every state to provide adequate means toward assessment and remediation of reading problems, not because of purported brain faults but because of the poor reading itself (Protopapas and Parrila, 2018, 2019).

To sum up, our concept of dyslexia, reflected in the evolution of definitions and discussions of the topic over the past decades, seems to be moving from a kind of natural category, to that of an educational label. That is, dyslexia is not some objective physical condition that occurs naturally and inherently characterizes some children, regardless of their linguistic and educational context and of whether or not they ever try to learn to read. Rather, it is a label applied in the context of specific social circumstances, demands, and pressures. This does not change the fact that certain children have great difficulty learning to read; that this is because of how their brains are set up;^6^ that they need special support to be able to function adequately in a literate society; or that society must provide that support. But it does change how they are viewed and what is to be done.

So, in what follows I will consider the implications of these elements, in the same order, focusing on how they have changed or are changing, from the past to the future. Let us start with implications for research.

## Implications for Research

When dyslexia is defined by discrepancy from IQ, this effectively increases the average IQ of study participants and ignores poor readers with low IQ, resulting in unrepresentative samples for research, and possibly unrepresentative findings in domains other than reading. Current research practice has escaped from discrepancy criteria but has retained a criterion of at least average intelligence. This is aimed to increase the homogeneity of research samples and mainly to avoid criticism that some findings, beyond reading performance, might be attributed to low cognitive ability.

When dyslexia is characterized by diverse symptom lists, it is tempting to try to find unifying explanations, that is, common causes for all observed symptoms. But if the symptoms aren’t reliably associated with dyslexia then this search is counterproductive. By focusing on the only common element across children with dyslexia, that is, the difficulty in learning to read, research can concentrate on identifying the necessary set of underlying skills and development routes, rather than trying to account for assorted, anecdotal, or simply unreliable elements.

When dyslexia is thought to specifically affect decoding, research will naturally focus on reading accuracy and on the cognitive and linguistic skills underlying accurate decoding. This is largely an artifact of English being spoken in the countries in which research was better funded, because English is an outlier orthography in which learning to read accurately is difficult, in contrast to other European orthographies (Share, 2008). Fortunately, with the progress of cross-linguistic research, and with data now available on many languages and orthographies, it has become clear that development of fluency is also very important, and must be studied on its own in addition to accuracy (Wolf and Katzir-Cohen, 2001; Kuhn et al., 2010).

If dyslexia is thought to be a specific identifiable condition then it is natural to search for clear criteria to demarcate it, and to debate definitions including or excluding these criteria. This direction loses its force if dyslexia is considered continuous with the general population, because the research focus shifts to the characterization of performance profiles, to the establishment of reliable and unreliable features, and to the quantification of different effects and contributions. In other words, this is a shift from qualities to quantities.

Similarly, if we expect that dyslexia is caused by a specific factor, or set of factors, then we will naturally put our efforts into identifying this factor and its effects. This has occupied several productive groups for decades, and there is no end to the proliferation of theories about what the distinct causes of dyslexia are (see Elliott and Grigorenko, 2014, for discussion of some of them). However, if we view the unsuitability of a brain for learning to read not as a product of some specific factor but, rather, as the product of a complex developmental pathway, in which genetics and environment act and interact to produce a complex phenotype, then attention is shifted to understanding these developmental pathways, the ways in which they differ, and their sensitivity to different manipulations. This is a very different kind of research program, eschewing simplistic dichotomies between “dyslexic” and “non-dyslexic” groups and their – often uninformative and potentially misleading – average differences, in favor of studying the graded effects of a multitude of risk and protective factors over multiple levels (genetic, neural, cognitive, behavioral) and their associations and interactions across levels of reading skill, ages, environments, and generations (van Bergen et al., 2014).

As noted by Bishop (2015), “a genetic aetiology does not mean a condition is untreatable.” Even though it is important to recognize limitations set by genetics, a better understanding of how genes work can help us focus on addressing specific difficulties of specific persons in specific ways rather than applying blanket measures, and possibly in the future to be able to further customize and individualize our approach beyond what is behaviorally observable. Having a better view of the scope and extent of environmental influence, we can focus on maximizing the efficiency of interventions. Two important avenues in this regard involve (a) identification of reliable precursors (i.e., risk factors) that can support valid early screening, well before literacy instruction, and (b) the development of interventions with validated long-lasting effects. Both of these are exemplified in the Jyväskylä Longitudinal Study of Dyslexia, which has produced both leads related to precursors as well as an applied framework for early intervention addressing crucial skill domains (Lyytinen et al., 2008, 2015; Solheim et al., 2018). The importance of early screening and intervention is highlighted by empirical confirmation of the longstanding assumptions that (a) when it comes to intervention, earlier is better (Lovett et al., 2017), and (b) kindergarten training in the foundational domain of phonological awareness has clinically significant effects lasting through secondary education (Kjeldsen et al., 2019). In this context, further research can transcend monolithic – and possibly misguided – approaches to the causes and outcomes of reading failure and address the efficiency of behavioral assays and environmental changers down to the level of the individual.

In other words, research should stop trying to answer the question “what causes dyslexia?” and instead put more effort into understanding the observed range of reading development trajectories and the extent to which different factors affect learning to read in different ages, genetic and environmental contexts, and instructional situations. This presupposes a major shift in underlying assumptions in that reading failure shall not be conceived of as the result of some critical early failure but, rather, as the cumulative outcome of multiple, potentially interacting, risk and protective factors, which may affect the developmental and instructional “initial conditions” as well as growth rates and environmental interactions along multiply determined individual paths.

Along these lines, if dyslexia is seen as a disorder, then by definition something is abnormal, and our job as researchers is to find it, characterize it, and try to cure it. However, if difficulty in learning to read is seen as part of cognitive variability, just individual differences as usual, then our job is to study and to understand this variability, the dimensions of variability, its causes and correlates, without being on the look for something abnormal.

Finally, if dyslexia is thought to be a condition, inherent in some children, then it is some kind of natural distinct entity and it makes sense to ask what exactly it is. In contrast, if we think of dyslexia as an educational label, then there is no reason to study its essence. Rather, we can concentrate on the features and contexts associated with this label and focus our research on the effects of interventions.

Now let us turn to implications for remedial education.

## Implications for Remedial Education

If you define dyslexia as a discrepancy from IQ, then you exclude low-IQ children from receiving remedial services to ameliorate their poor reading. This is not only unethical, as you deprive children of the help they need; it is also incorrect, because, as noted above, there is no difference in reading intervention or in prognosis based on IQ. In contrast, if cognitive ability does not factor into your definition, then children can be eligible for remedial services on the basis of their reading performance, which is the only relevant factor.

If you define dyslexia as a constellation of assorted symptoms, then you may waste time, efforts, and resources, in assessing – and perhaps addressing – various irrelevant domains, for example, fine motor coordination, balance, auditory skills, or general visual or oculomotor skills. In contrast, if your concept of dyslexia focuses on reading skill, then your assessment and intervention will also focus on reading skill. Let me clarify here that I do not imply that only reading should ever be assessed, or that only reading skills should be practiced, for two reasons. Although only reading is relevant for *diagnosing* dyslexia (de Jong and van Bergen, 2017), much more is needed when it comes to *intervention*. First, a wider cognitive and learning profile may be necessary for the special educator or educational psychologist, to identify the weak and strong areas for any given child, because the individualized educational plan will have to be based on the strengths and build from them to address the weaknesses, including weaknesses in reading and possibly others, thereby comprehensively addressing the needs of the child. And second, reading is itself dependent on certain linguistic and meta-linguistic skills and prerequisite knowledge (Vellutino et al., 2004; Melby-Lervåg et al., 2012). So, the specialist will consider and may have to support the development of phonological processing, phonological memory, phonological awareness, knowledge of the alphabet, and conventions of print, before reading itself can be addressed. This is not because dyslexia is thought to be an assortment of disconnected clumsiness, but because reading skills are documented by research to build on prerequisite skills and, in turn, to form the basis for additional skills to build on them later.

If you define dyslexia as an impairment in decoding, then you will focus intervention on decoding, and you will consider your job done when the child reads accurately. But your job is not done, because the child may read so slowly as to be functionally illiterate (cf., Torgesen, 2005). Word reading speed can only begin to develop after high accuracy has been achieved, while attainment of reading fluency requires additional skills beyond isolated word reading speed (Juul et al., 2014; Altani et al., 2019). Thus, word-level skills must build to an efficiency level that can sustain reading to learn. By acknowledging this wider concept of word-level reading, and consequently by allowing low fluency to factor in the concept of dyslexia, your assessment and intervention efforts will naturally include fluency as an assessment domain and remedial target, leading to better functional outcomes.

If you think of dyslexia as a discrete identifiable condition then you will seek to apply clear criteria to determine who should receive remedial services, and you will expect unambiguous classification from your criteria. You will think your criteria are incomplete or incorrect when they fail to meet this standard. However, if you admit that dyslexia is continuous with the general population then gray areas and ambiguities are inherently expected. Therefore your assessment and your remedial goals will be accepted for what they are, namely multi-factorial choices, where some things may not be very clear or may be treated differently in different contexts. This requires the exercise of more judgment on behalf of the clinician; but this is why we need expert, well-trained clinicians, so they can exercise their judgment wisely and toward the benefit of the children under diverse circumstances and resource pressures.

If you think dyslexia is caused by some factor X, and if you think you know what X is, then you will probably try to fix X, expecting reading to improve as a result, instead of addressing the reading difficulties directly. This kind of thinking is still very much alive. However, if the trend is away from single causes and toward the recognition that reading skill, and reading failure, is multi-factorial, multi-level, and polygenic, then you are more likely to recognize that assessment and remedial efforts are best focused directly on reading skill and the well-known prerequisites for its development.

If you think that dyslexia is deterministically caused by the genes and fixed at birth, then you can either be searching for treatment, in the medical sense (opening the field to multiple contenders for miracle cures); or you can see your role as merely providing support so that one’s life can be led in spite of the reading handicap. In this approach, it matters little when the treatment or the support is provided, since the problem is fixed. If, on the other hand, you see the poor reading as a long-term developmental outcome of specific propensities expressed in specific environments, then your role will be drastically different, as you will seek to identify the trajectories leading to unfavorable outcomes as early as possible, and to apply corrective environmental measures that can lead to new trajectories and, hence, to better outcomes. Crucially, the appreciation of the true scope afforded by environmental factors can help focus efforts on early screening and early intervention to the largest extent possible, and tailored to the specific needs of each individual, thus maximizing the long-term benefits for each person and the overall social impact from a wider perspective.

Related to the previous point, if you think of dyslexia as a neurodevelopmental disorder, that is, a kind of physical or mental disease, then you may see children with difficulty learning to read as being “disordered,” “afflicted,” and “impaired.” You will think of them as having “a condition.” But if you accept that difficulty in learning to read is part of normal variability, as can be difficulty in learning any other thing, then you will not think of poor readers as having a disease. Rather, you will see them as having strong and weak areas, just like all children, and you will build on the strengths to address the weaknesses, helping them to achieve their potential in the context of the societal demand for universal literacy.

In other words, if dyslexia is considered to be a natural category, then children with dyslexia will be largely treated as aliens, since they are of a different kind, afflicted with some undesirable condition. If, on the other hand, dyslexia is recognized as a label for the low end of reading skill, part of normal individual variability, then decisions about assessment and interventions can be guided by richer considerations, including cultural context, expectations, attitudes, and so on. Although some heavy ethical issues can be involved in these kinds of discussions, I don’t think they can be avoided, or that they should be avoided. Societies that value universal literacy must deal comprehensively with the side-effects of the pressures they apply.

Crucially, this shift in viewpoint does not in any way imply that reading difficulties should be left unexamined or untreated. My intended message is in fact quite the opposite: given the potential adverse consequences of poor literacy as a source of social inequality, it is critical that risk for reading failure is detected and ameliorated as much and as early as possible. Although the research is nowhere near there yet, it is conceivable and desirable that, in the future, risk of reading failure will be reliably detected in preschool, and possibly prevented before reading instruction. For the time being, adequate remedial support must be provided throughout school age to every child with poor prognosis for literacy outcomes, in order to minimize the negative downstream effects on reading difficulties. The point I want to make here is that intervention is not to be provided because of a presumed abnormality or discrete pathological condition, but because of the poor reading itself, in the context of the literate society in which we live.

## Conclusion

Table 2 concisely displays the aforementioned conceptual elements of definitions of dyslexia and their corresponding implications for research and remedial education, from past to future. In closing, perhaps another way to state this conceptual evolution more generally is as a transition from considering dyslexia to be some entity that *causes* poor reading, toward considering the term dyslexia to simply label poor performance, as a *name* for poor reading. Specifically, dyslexia is a name for “persistent and unexpected difficulty in developing age- and experience-appropriate word reading skills” (Parrila and Protopapas, 2017, p. 333; this excludes cases of primary difficulties with understanding the text, which are best conceived as a different type of problem; Catts et al., 2006; Nation and Angell, 2006). This conceptual shift has great implications for research, as we have seen, as it renders obsolete any searches for elusive abnormalities, and directs efforts toward understanding reading skill as a multifaceted domain following a complex, multifactorial developmental course. Some of these developmental courses underlie effortless and efficient learning to read, but others much less so. For education, the conceptual shift directs educators, as well as parents and children, to stop thinking of dyslexia as something abnormal that hinders their reading, or as a diagnosed medical condition, and instead to embrace individual variability, assess relevant skills, and build on strengths to address the weaknesses in the developmentally appropriate sequences. The most important goal in this endeavor would be to minimize the negative impact of reading difficulties on individuals’ life quality and overall social equality, away from outdated and counterproductive conceptualizations and toward modern approaches that embrace and address the complexities of both mind and education.

**TABLE 2:** Elements of dyslexia definitions, and their implications for research and remedial education, in the past and future.

**Conceptual element of definition**		**Implications for research**		**Implications for remedial education**	
		
**Past**	**Future**	**Past**	**Future**	**Past**	**Future**
Discrepancy from IQ	Across ability range	Ignore children below average IQ	Include at least low average IQ	Exclude low-IQ cases	Include all poor readers
Diverse symptom list	Word-level processing	Seek common causes	Focus on reading skill	Highlight and left suboptimal functions	Concentrate on reading-related profile
Single word decoding	Word reading difficulty	Measure accuracy	Accuracy and fluency	Set decoding targets	Also work on fluency
Discrete identifiable condition	Continuous with the general population	Define and debate diagnostic criteria	Characterize performance profiles	Clear cut-off criteria	Multi-factorial choices
Caused by X (factor accounting for all)	Multifactorial, multi-level, polygenic	Search for distinct cause(s)	Seek developmental pathways	Try to fix X (expect reading to improve)	Focus on reading, build on strengths
Determined by genes; fixed at birth	Interplay of multiple interacting factors	Seek cures or look for support potential	Study environmental changers	Try to cure or to circumvent; any time	Change early conditions to improve outcomes
Neurodevelopmental disorder	Within normal variability	Look for abnormality	Study variability	Label as disordered, afflicted, impaired	Identify weak areas; provide support
Natural category	Educational label	Ask what dyslexia is	Ask how to help	Treat as different kind	Contextualize and guide

## Author Contributions

The author confirms being the sole contributor of this work and has approved it for publication.

## Conflict of Interest

The author declares that the research was conducted in the absence of any commercial or financial relationships that could be construed as a potential conflict of interest.

## Footnotes

1 See Peterson and Pennington (2015) for a discussion of dyslexia from a clinical perspective.

2 http://www.merriam-webster.com/dictionary/disorder

3 http://medical-dictionary.thefreedictionary.com/disorder

4 http://www.oxforddictionaries.com/definition/english/disorder

5 http://www.dictionary.com/browse/disorder

6 Note that there is a subtle but crucial difference here: on the one hand, one fails to learn to read (despite adequate motivation, opportunity, and instruction, in a given social and educational environment) largely because of how their brain is set up – and in this sense dyslexia is objective and biological. But, on the other hand, dyslexia is not the condition of the brain or the cause of the failure to learn to read; rather, it is the outcome of (unsuccessfully) trying to learn to read in the specific circumstances of the given environment. Dyslexia cannot exist in an illiterate society even though people can exist there who might fail to learn to read in specific literate societies (but possibly not in others).
